# Gelation and Re-entrance in Mixtures of Soft Colloids and Linear Polymers of Equal Size

**DOI:** 10.1021/acs.macromol.2c02491

**Published:** 2023-02-22

**Authors:** Daniele Parisi, Domenico Truzzolillo, Ali H. Slim, Phillippe Dieudonné-George, Suresh Narayanan, Jacinta C. Conrad, Vishnu D. Deepak, Mario Gauthier, Dimitris Vlassopoulos

**Affiliations:** † FORTH, Institute of Electronic Structure and Laser, Heraklion 70013, Crete, Greece; ‡ Department of Materials Science and Technology, University of Crete, Heraklion 70013, Crete, Greece; § Department of Chemical Engineering, Product Technology, University of Groningen, Nijenborgh 4, Groningen 9747 AG, The Netherlands; ∥ Laboratoire Charles Coulomb (L2C), UMR 5221 CNRS Université de Montpellier, Montpellier 34095, France; ⊥ Department of Chemical and Biomolecular Engineering, University of Houston, Houston, Texas 77204-4004, United States; # Advanced Photon Source, Argonne National Laboratory, Argonne, Illinois 60439, United States; ¶ Department of Chemistry, University of Waterloo, Waterloo, Ontario N2L 3G1, Canada

## Abstract

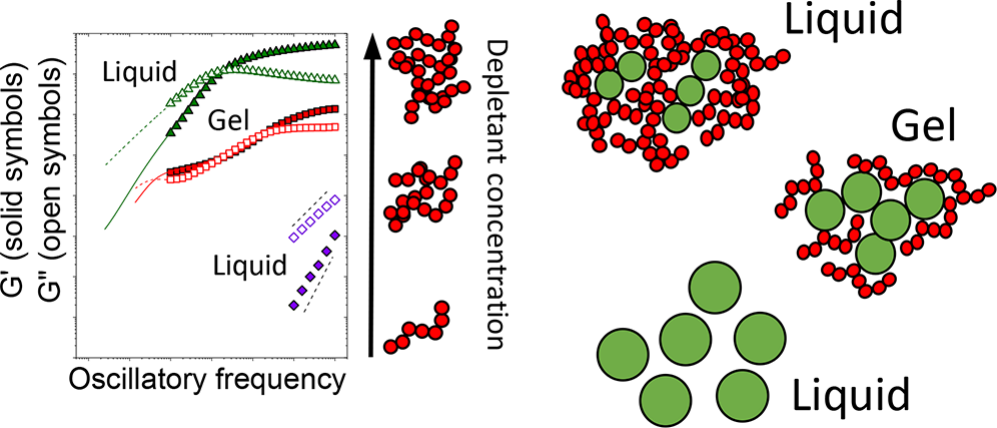

Liquid mixtures composed of colloidal particles and much smaller non-adsorbing linear homopolymers can undergo a gelation transition due to polymer-mediated depletion forces. We now show that the addition of linear polymers to suspensions of soft colloids having the same hydrodynamic size yields a liquid-to-gel-to-re-entrant liquid transition. In particular, the dynamic state diagram of 1,4-polybutadiene star–linear polymer mixtures was determined with the help of linear viscoelastic and small-angle X-ray scattering experiments. While keeping the star polymers below their nominal overlap concentration, a gel was formed upon increasing the linear polymer content. Further addition of linear chains yielded a re-entrant liquid. This unexpected behavior was rationalized by the interplay of three possible phenomena: (i) depletion interactions, driven by the size disparity between the stars and the polymer length scale which is the mesh size of its entanglement network; (ii) colloidal deswelling due to the increased osmotic pressure exerted onto the stars; and (iii) a concomitant progressive suppression of the depletion efficiency on increasing the polymer concentration due to reduced mesh size, hence a smaller range of attraction. Our results unveil an exciting new way to tailor the flow of soft colloids and highlight a largely unexplored path to engineer soft colloidal mixtures.

## 1 Introduction

Colloidal mixtures have emerged as model systems to tailor the phase behavior and flow properties of suspensions, with implications extending from fundamental understanding^1−12^ to ubiquitous applications.^13−17^ It has been 20 years since the seminal work of Pham et al.^18^ highlighted the effect of depleting agents on concentrated hard sphere suspensions and more than 60 years since Asakura and Oosawa^19^ unveiled the entropic nature of the attractions between colloids in the presence of smaller particles.

Model hard sphere colloidal suspensions and their mixtures have been intensively studied, whereas the effects of particle softness have been less explored. When a soluble polymer is added to a colloidal suspension, the interactions between colloidal particles are modified, and the macroscopic response of the suspension can change dramatically. For example, a transient colloidal network may be formed at low particle volume fractions, below the particle concentration where the dynamics freeze and glass transition occurs. In this case, gelation (or flocculation) is driven by forces of entropic nature, namely, depletion forces. This phenomenon has been observed in a variety of mixtures, with polymers or colloids playing the role of depletants, and hard or soft particles experiencing depletant-mediated attractions.^1,5−7,9,11,20−25^ The depletant-to-depleted particle size ratio determines the attraction range, and the depletant concentration determines the attraction strength between a pair of particles.^19,26^ This scenario holds for colloids whose internal microstructure and shape are not affected by the addition of depleting agents, for example, hard spheres. However, for soft colloidal particles, whose microstructure depends on the osmotic pressure of the surrounding medium,^27,28^ the precise state diagram is currently unknown, and whether a depletion gel state persists upon increasing the depletant concentration (attractions) remains an open question.^29^

Star polymers^1,4,5,7−10,30^ represent excellent model systems for soft colloids, thanks to their tunable softness dictated by the branching functionality and the degree of polymerization of their arms.^21^ In addition, despite the demanding synthesis, they are relatively simple in the sense that they consist of homopolymer arms without enthalpic effects, they can span the entire concentration range from solution to the melt, and their dynamics are reasonably well understood. Recently,^31^ we addressed the transition from confined-to-bulk dynamics of linear homopolymers added to non-dilute star-polymer solutions, with linear and star polymers having nearly the same hydrodynamic size. Till today, emphasis was placed on problems involving star polymers well above their overlap concentration, typically in the glassy state. In the present work, by using the same systems as Parisi et al.,^31^ we investigate the dynamic state diagram of star polymers in a good solvent, below their overlap concentration, upon addition of linear polymer chains. We address in particular, three fundamental questions: what is the consequence of adding linear polymer chains to a liquid-like star-polymer suspension at fixed number density and equal hydrodynamic size? How does the interplay between osmotic shrinkage and depletion (mediated by the size of the depletant) control the dynamics of the mixtures? Does the scenario encountered for hard-sphere (HS)–polymer mixtures, with a unique liquid-to-gel transition, still hold?

We found that, starting from a solution of star polymers of different softness (functionality) and slightly below their overlap concentration (C*), the addition of linear polymers of equal size drives the system to a dynamic arrest. Such an arrested state is the result of depletion forces exerted on the stars and ruled by a characteristic correlation length of the order of the mesh size of the formed topological chain network. A further increase of the concentration of linear chains promotes a re-entrant liquid state, whose rheological response is mediated by the linear polymer content. This novel re-entrance is further corroborated by the behavior of the mixtures at lower star concentrations. Indeed, the progressive addition of linear polymers yields: (i) first a critical gel, which is followed by typical solid-like (network) behavior with detectable structural relaxation, (ii) a correlated amorphous liquid (exhibiting liquid-like order) with an observable relaxation dictated by the center-of-mass motion of the stars (colloidal mode), and (iii) a viscoelastic liquid whose response is dominated by the contribution of the polymeric network. Small-angle X-ray scattering (SAXS) experiments supported the scenario of star deswelling, due to the stars themselves (packing effect) and/or the increasing concentration of linear chains (osmotic shrinkage). At the same time, the reduced polymer network mesh size upon increasing concentration indicates that a smaller depletant size leads to a smaller attraction range, corroborating the existence of a (metastable) liquid state due to the progressive suppression of the depletion efficiency.^32−34^

## 2 Experimental Section

### 2.1 Materials

We used two multiarm 1,4-polybutadiene (PBD) stars, identified as S362 and S1114, with number-average branching functionality, weight-average arm molar mass, and respective polydispersity equal to *f* = 362 arms, *M*_w_^a^ = 24400 g/mol, *M*_w_/*M*_n_ = 1.06, and *f* = 1114 arms, *M*_w_^a^ = 1270 g/mol, *M*_w_/*M*_n_ = 1.06.^35^ Both stars were used in previous work, with the high functionality star S1114 being considered as a nearly hard colloidal particle.^7,36,37^ Relevant details on the synthesis and the size exclusion chromatography analysis of these samples are reported elsewhere.^20,35^ According to the well-known Daoud–Cotton model,^38^ a star polymer in a good solvent is characterized by a non-homogeneous monomer density distribution that comprises three regions: an inner melt-like core, an intermediate ideal region, and an outer excluded volume region. The latter is involved in interactions with neighboring stars in crowded suspensions.

Two PBD linear polymers, identified as L1000 and L243, were also used. L1000 was obtained from Polymer Source (Canada) and has a weight-average molar mass *M*_w_ = 1,060,000 g/mol and *M*_w_/*M*_n_ = 1.1. L243 was provided by Prof. N. Hadjichristidis (KAUST) and has *M*_w_ = 243,000 g/mol and *M*_w_/*M*_n_ < 1.1.

The polymers were dissolved in squalene, a non-volatile solvent providing good (nearly athermal) solvency conditions for PBD.^39^ The hydrodynamic radius was determined with dynamic light scattering (DLS) measurements under dilute conditions. The hydrodynamic radii (*R*_H_) and overlap concentrations (C*) for all the investigated samples are reported in Table 1. The DLS characterization of L243 at 20 °C, illustrated in Figure S1 of the Supporting Information, yielded *R*_H_ = 15.3 nm and *C*_L_^*^ = 27 mg/mL. The star–linear polymer mixtures S352/L1000 are characterized by a hydrodynamic size ratio equal to 39/41 = 0.95, whereas the respective mixtures of hard-like spheres and linear polymers S1114/L243 have a ratio of 12/15.3 = 0.78. The nearly identical size of the stars and linear chains, as well as the different star softness, are responsible for the unusual and rich dynamic state diagram presented in this work. The molecular characteristics of the polymers, together with the light scattering results, and the volume fraction at the glass transition for the stars, ϕ_g_, taken from previous work^7,31,37^ are summarized in Table 1. The mixtures were always prepared starting from a pure star-polymer solution below ϕ_g_. When linear polymer chains were added, the same star volume fraction ϕ_s_ = *C*/C* was maintained, so that the linear chains did in fact replace part of the solvent. To distinguish it from the soft stars, the volume fraction of the hard-like colloids, S1114, will be hereafter identified as ϕ_HS_. The linear polymer concentration, in both mixtures and pure solutions, is expressed as the nominal concentration (mg/mL) of chains excluding the stars, that is, , where *W*_L_ and ρ_L_ are the mass and the density (892 mg/cm^3^) of the dissolved linear polymer chains and *V*_sol_ is the volume of the solvent (squalene).^31^

**Table 1:** Molecular Characteristics of the Star and Linear Polymers

code	*M*_w_ [kg/mol]	*M*_w_^a^ [kg/mol]	*f* [-]	*R*_H_^a^ [nm]	C*(or*C*_L_^*^)^b^ [g/mL]	*C*_g_^c^ [g/mL]	ϕ_g_ = *C*/*C*_g_ [-]
S1114	1415	1.27	1114	12.0 ± 0.5	0.326	0.26–0.30	0.8–0.92
S362	8832	24.4	362	39.0 ± 2	0.062	0.09–0.12	1.5–2
L1000	1060			41.0 ± 0.8	0.006		
L243	243			15.3 ± 0.5	0.027		

a Estimated from the diffusion coefficient *D*, measured with DLS in squalene at 20 °C, at a concentration 1 mg/mL, in the dilute regime.

b The overlap concentration was estimated as .

c The glass transition was estimated from the rheological experiments in the linear viscoelastic regime. A suspension not exhibiting terminal relaxation within the frequency range 0.01–100 rad/s was technically considered a glass.^1,5,7,31,37^

The linear chains in the mixtures were always entangled. The entanglement volume fraction of the linear chains can be estimated as  for good solvents,^40^ where *M*_e_ is the entanglement molar mass (1850 g/mol^41^). This yields ϕ_e_ = 0.008 and ϕ_e_ = 0.24 for L1000 and L243, respectively. The lowest linear polymer volume fractions (ϕ_L_ = *C*_L_/*C*_L_^*^, with *C*_L_ being the concentration of linear chains) probed in this work were ϕ_L_ = 3 and ϕ_L_ = 1.1 for L1000 and L243, respectively. The characteristic length of an entangled linear polymer matrix is given by the correlation length ξ = , with *kT* being the thermal energy, and *G*_N_^0^(ϕ) = *G*_N_^0^(ϕ = 1)ϕ^2.3^ (*G*_N_^0^ (ϕ = 1) = 10^6^ Pa^41^), the diluted polymer plateau modulus.^40^ The correlation length ξ can be thought of as the average spatial distance between neighboring entanglement points and is the length dictating the range (and the magnitude) of depletion interactions in semidilute solutions.^34,42^ All the experiments were performed at 20 °C.

### 2.2 Rheology

The dynamics of the star–linear polymer mixtures were investigated with rheological measurements, which were performed using a sensitive strain-controlled rheometer (ARES-HR 100FRTN1 from TA, USA). Due to the very limited amounts of samples available, a small home-made cone-and-plate geometry (stainless-steel cone with 8 mm diameter, 0.166 rad cone angle) was mostly used. At very low concentrations, a 25 mm stainless-steel cone (with angle 0.02 rad) was used to increase the torque signal. The temperature was set to 20.00 ± 0.01 °C and controlled using a Peltier plate with a recirculating water/ethylene glycol bath. During an experimental run, the sample (which had a pasty appearance) was loaded on the rheometer, with special attention to avoid the appearance of bubbles, and a well-defined pre-shear protocol was applied such that each sample was subjected to: (i) a dynamic strain amplitude sweep at fixed frequency (100 rad/s) to determine the linear viscoelastic regime, that is, where the moduli did not show any detectable dependence on strain amplitude; (ii) a dynamic time sweep at large nonlinear strain amplitude (typically 200%) and low frequency (1 rad/s), to effectively shear-melt (i.e., rejuvenate) the sample, as judged by the time-independent first harmonics *G*′(ω,γ_0_) and *G*″(ω,γ_0_) (this step typically lasted 300 s); (iii) a dynamic time sweep for a (waiting) time *t*_w_ ≈ 10^5^ s, which was performed in the linear regime to monitor the time evolution of the moduli to steady state, corresponding to an aged sample; and (iv) small-amplitude oscillatory shear tests in the frequency range 100–0.01 rad/s, to probe the linear viscoelastic spectra of the aged samples.

It is worth pointing out that the rejuvenation induced by pre-shearing the samples at amplitudes deeply into the nonlinear regime (γ_0_ > 200%) erases the accumulated aging. Based on the available experimental evidence, the eventual steady state, typically characterized by a viscoelastic response [*G*′(ω), *G*″(ω)] independent of the waiting time for more than 12 h,^31^ did not depend on the details of the rejuvenation protocol (the responses of the aged samples did not show any detectable dependence on the preshear amplitude γ_0_ > 200% and frequency in the range 1 rad/s ≤ ω ≤ 10 rad/s). This makes the adopted procedure robust, and it has been widely discussed in many of our previous studies.^6,20,31,37,43^

Additionally, creep experiments were performed to extend the low-frequency region of the oscillatory response. A stress-controlled rheometer MCR 501 (Anton-Paar, Austria), equipped with a stainless-steel cone-plate geometry (8 mm diameter, cone angle of 0.017 rad), was used for creep measurements. The temperature was controlled by a Peltier element that also constituted the lower plate. Different stresses were applied to ensure that the response reflected the linear viscoelastic regime. Creep compliance was then converted into dynamic moduli by means of the nonlinear regularization method proposed by Weese^44^ (see the Supporting Information for further details).

It should be noted that when these suspensions were out of equilibrium, they exhibited time-dependent dynamics (aging)^31,45−47^ which was taken into account. Typical aging time was between 10^3^ and 10^5^ s, depending on the suspension concentration. The data shown hereafter refer only to aged samples, and the influence of aging will not be discussed further. For ϕ > ϕ_g_, the star-polymer suspensions behave as viscoelastic solids, with both storage (*G*′) and loss (*G*″) moduli weakly dependent on frequency, *G*′ > *G*″ and *G*″ exhibiting a shallow, broad minimum typical of glassy colloids.^47−51^

### 2.3 Small-Angle X-ray Scattering

SAXS measurements were performed with an in-house setup (Montpellier). A high-brightness, low-power X-ray tube, coupled with aspheric multilayer optic (GeniX^3D^ from Xenocs) was employed. It delivers an ultralow divergent beam (0.5 mrad, λ = 0.15418 nm). Scatterless slits were used to give a clean 0.6 mm beam diameter with a flux of 35 Mphotons/s at the sample. We worked in a transmission configuration, and scattered intensity was measured using a 2D “Pilatus” 300K pixel detector by Dectris (490 × 600 pixels) with pixel size (area) of 172 × 172 μm^2^, at a distance of 1.9 m from the sample loaded in cylindrical quartz capillary tubes (Hilgenberg, 1 mm diameter). SAXS data were collected across a scattering wavevector range 0.07 nm^–1^ < *q* < 0.2 nm^–1^. The temperature was kept fixed (*T* = 20.0 ± 0.1 °C) via a recirculating water/ethylene glycol bath. All intensities were corrected by transmission, and the empty cell contribution was subtracted.

For the star–linear polymer mixture with ϕ_s_ = 0.83 and *C*_L_ = 30 wt %, the SAXS measurements were performed at the beamline 8-ID-I in the Advanced Photon Source at Argonne National Laboratory (USA). The sample was loaded into a Quartz capillary tube (Charles-Supper, 2 mm inner diameter) and then sealed using wax to prevent solvent evaporation. SAXS data across a wavevector range 0.01 nm^–1^ < *q* < 0.3 nm^–1^ were collected and corrected according to the background scattering of each sample. A copper block with a Peltier plate was used to control the temperature. The captured scattering intensities were processed using a GUI visualization software package that was developed and provided by APS.

## 3 Results and Discussion

Before presenting the results obtained with the star–linear mixtures, we report on the structural and rheological features of the (reference) pure star system in a good solvent, at different star concentrations. These experiments were crucial for the subsequent study of the mixtures since they allowed us to locate the liquid-to-glass transition of the stars, as well as to inspect structural changes due to the increase of colloid number density.

**Figure 1:**
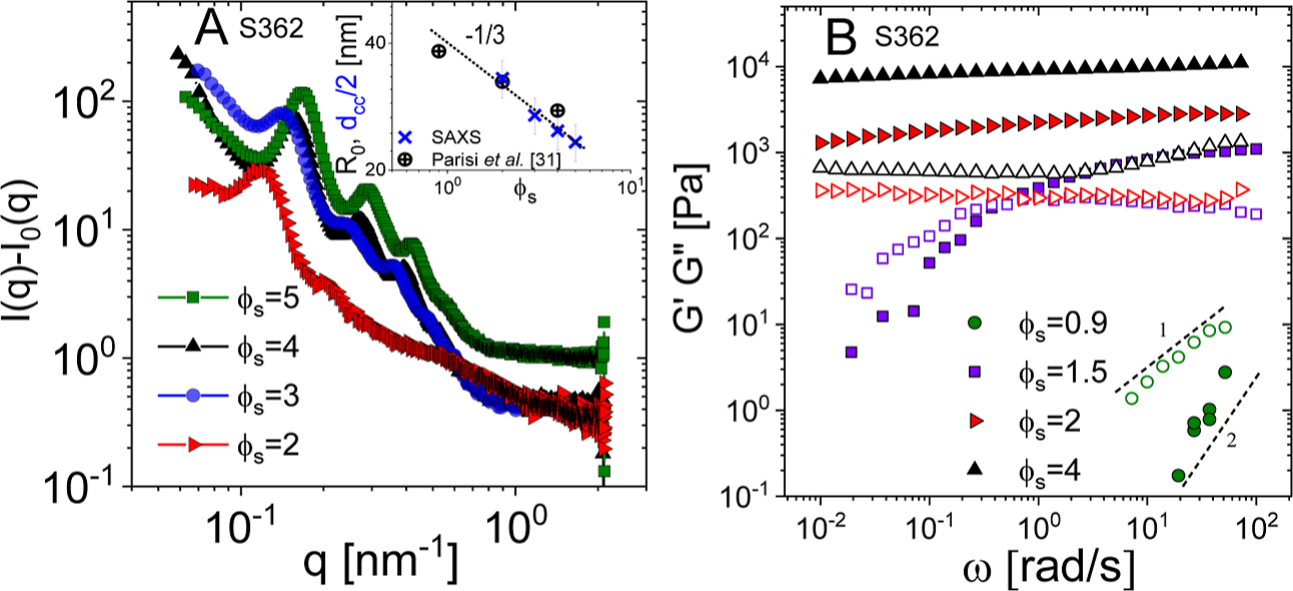
(A) SAXS intensity [*I*(*q*) – *I*_0_(*q*)], with *I*_0_(*q*) being the background intensity, for pure S362 star-polymer solutions in squalene at various volume fractions ϕ_s_ (see the legend) as a function of the scattering wavevector *q*. Inset: star radius (*R*_0_) and average half distance between cores (*d*_cc_/2) as a function of the star volume fraction (ϕ_s_). The open circles are calculated values from a study by Parisi et al.,^31^ whereas the × symbols are estimated as π*a*/*q*_max_ where *q*_max_ is the scattering wavevector at the low-*q* intensity peak, and the coefficient a, set to 1.23, reflects a structure controlled only by two-body correlation.^52^ The dashed line represents the (−1/3) power-law determined from the SAXS data. (B) Storage modulus *G*′ (solid symbols) and loss modulus *G*″ (open symbols) as a function of the oscillation frequency ω for pure S362 stars in squalene at various volume fractions ϕ_s_ (see the legend) from a study by Parisi et al.^31^ The dashed lines highlight the terminal slopes (see text). All solutions were measured after steady-state conditions were reached (aging between 10^3^ and 10^5^ s).

In Figure 1A, we report the SAXS intensity *I*(*q*) after background (empty cell) subtraction, as a function of the scattering wavevector *q* for the pure S362 star suspensions at different concentrations 2 ≤ ϕ_s_ ≤ 5, all above the glass transition. We observe correlation peaks due to the amorphous dense packing of the stars (liquid-like order). The *I*(*q*) peaks shift to larger *q* values as the concentration is increased, pointing to a reduction of the average distance between the scatterers, here the silicon-rich core of the stars. It is possible to determine the average distance between the cores as *d*_cc_ = 2π*a*/*q*_max_, where *q*_max_ is the scattering wavevector at the first low-*q* peak of the scattered intensity and *a* = 1.23 is a numerical prefactor reflecting a structure controlled by two-body correlation.^52^ We found excellent agreement between the measured half distance *d*_cc_/2 and the star radius computed on the basis of osmotic theory^5,26,30,53^ (see Table S1 in the Supporting Information and the inset in Figure 1A).^31^ We found that *d*_cc_/2 exhibits a scaling dependence on star concentration which is compatible with that of concentrated and homogeneously distributed scatterers (*d*_cc_ ∼ ϕ_s_^–1/3^^54^), not very different from the ϕ_s_^–1/5^ power-law estimated in our previous work for the star radius.^31^ More remarkably, the very good agreement between the *d*_cc_/2 and *R*_0_ values supports the fact that interpenetration between the stars is very limited and that deswelling due to presence of neighboring stars is more severe than that for linear chain solutions, *R*(*C*_L_) ≈ *C*_L_^–1/8^. Since the osmotic pressure increases with functionality *f*,^55,56^ the stars are more efficient “osmotic compressors” than linear chains.^31^

The rheological spectra including the storage (*G*′) and loss (*G*″) moduli as a function of oscillatory frequency ω for pure S362 at various volume fractions (ϕ_s_), from below to well above the glass transition, are shown in Figure 1B. At ϕ_s_ = 0.9, the S362 suspensions exhibit a response typical for a (viscoelastic) liquid, with *G*″(ω) ≫ *G*′(ω) and terminal frequency scaling of 1 and 2, respectively. For 1.5 < ϕ_s_ < 2.0, the glass transition takes place, and for ϕ_s_ > 2.0, the stars, significantly deformed, attain the so-called jammed glass state (see Chapter 6 of ref (27)).

In this respect, it is important to specify that here we consider the star deformation to be isotropic and we neglect the possible onset of faceting that may occur at high volume fractions. In addition, as the arm segments in the outer corona region are free to move and rearrange compatibly with their excluded volume, we conjecture that faceting is reduced, if not absent, especially for ϕ_s_ < 1, where stars preferentially slightly interpenetrate rather than deform. Moreover, all our SAXS results are compatible with the deformation computed, assuming an average isotropic compression [Figure 1A (inset) and Figure S5 in Supporting Information].

**Figure 2:**
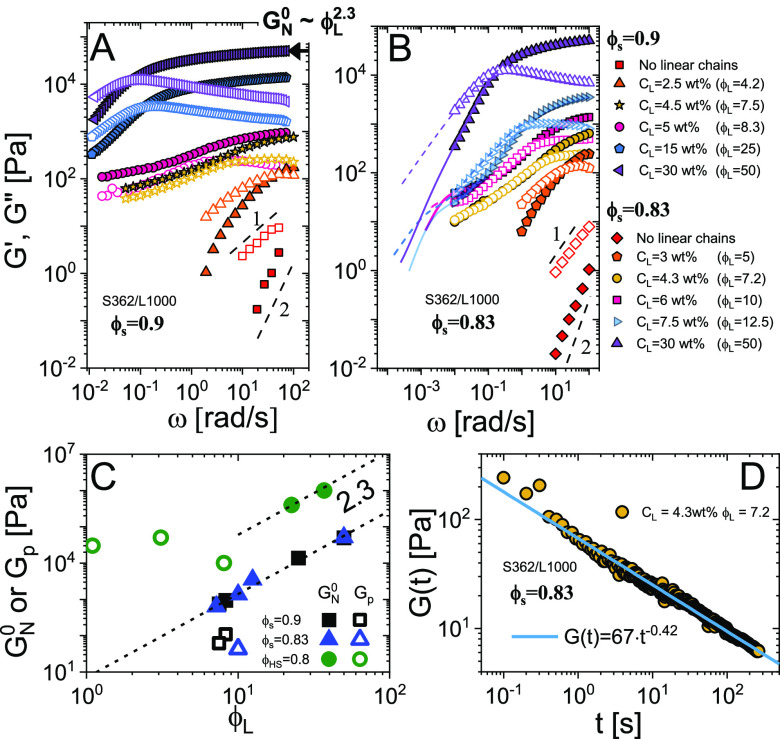
Linear viscoelastic spectra in terms of *G*′ (solid symbols) and *G*″ (open symbols) as a function of the oscillation frequency ω for S362-L1000 mixtures at various concentrations of L1000 (see the legends) and fixed star volume fractions: ϕ_s_ = 0.9 (A) and ϕ_s_ = 0.83 (B). Data at ϕ_s_ = 0.5 and ϕ_s_ = 0.7 are reported in the Supporting Information (Figure S4). The horizontal black arrow in panel (A) indicates the diluted plateau modulus (*G*_N_^0^) of a pure linear chain solution at 30 wt % (see the text). Solid and dashed lines in panel (B) are creep data converted to dynamic moduli (see Figure S2 of the Supporting Information). The dotted lines with slopes 1 and 2 indicate a fully relaxed viscoelastic fluid. (C) Polymeric (*G*_N_^0^) (solid symbols) and colloidal (*G*_p_) (open symbols) plateau moduli as a function of the linear chain volume fraction ϕ_L_. The dashed lines report the power-law dependence of entangled linear polymer chains in a good solvent.^40^ (D) Stress relaxation modulus *G*(*t*), obtained from a step-strain experiment, for *C*_L_ = 4.3 wt % as a function of time. The solid line represents the best fit to a power-law function *G*(*t*) = 67*t*^–0.42^ (see the text). The strain amplitude applied for the step-strain test was 1%, well within the linear viscoelastic regime. All the solutions were measured after steady-state conditions (aging between 10^3^ and 10^5^ s).

The addition of linear chains, as detailed hereafter, has a radically different impact on the star suspensions, compared with that of simple star crowding. Figure 2A shows the LVE response in terms of the frequency-dependent *G*′ and *G*″ for a pure star-polymer S362 solution at ϕ_s_ = 0.9 and its mixtures with linear chains L1000. The S362 solutions exhibit the typical behavior of a fully relaxed viscoelastic liquid (Figure 1B). As the linear polymer concentration is increased in the 4.5–5 wt % range, the linear viscoelastic spectra exhibit a liquid-to-solid transition. In this case, where the colloid–polymer interactions are purely repulsive, a depleting layer with a thickness proportional to the polymer correlation length ξ forms around each sphere and a depletion attraction occurs between colloids.^57^ In other words, the system can be thought of as soft colloids suspended in a sea of uncorrelated polymeric blobs of size ξ. Hence, the size ratio that should be considered for depletion effects is the one between the hydrodynamic size of the stars (R_H_ = 39 nm) and the entanglement distance ξ.^57^ For instance, if we consider the mixture in Figure 2A, with L1000 at 5 wt % (circles in Figure 2A), a correlation length of 16 nm is obtained, corresponding to a size ratio ξ/*R*_H_ ∼ 0.4, which represents a sufficient condition for depletion effects.^5,8^ Therefore, a star-polymer suspension undergoes gelation upon addition of an entangled network of linear homopolymer chains having nearly the same hydrodynamic radius.

Quite strikingly, a further increase in the linear chain concentration leads to a re-entrant liquid state (see data at *C*_L_ = 30 wt % in Figure 2A). The conditions under which we find such an unexpected multiple transition are different as compared with those reported in previous work on star–linear homopolymer mixtures.^5,20^ This is mainly due to two reasons: (i) the initial star-polymer suspension is a viscoelastic liquid, whereas previously ϕ_s_(ϕ_L_ = 0) > ϕ_g_ and (ii) the hydrodynamic size of the stars and the linear chains is nearly identical. In ref (20), the addition of small linear chains (with ) to a depleted star-polymer glass yielded a re-entrant viscoelastic solid that was identified as a gel, whereas in ref (5), the addition of large linear chains to a star glass (with ) did not promote any solid-to-liquid transition, even at large linear polymer chain fractions. We also note for completeness that in large hard sphere–small linear polymer mixtures, the attractive glass is established as a re-entrant state, resulting from the continuous addition of polymers to the depleted repulsive glass.^18,58^ In the present work, a gel is promoted by depletion, while the further addition of linear chains results in a re-entrant liquid. We attribute such a re-entrant liquid transition to the continuous action of osmotic forces mediated by the linear polymer chains. Such osmotic forces are responsible for two distinct effects when the concentration of linear chains is increased: (i) they cause the deswelling of the stars^31^ and (ii) they result in a reduced spatial range of depletion attractions because of the mesh size reduction of the semidilute polymers.^32−34^ These two effects can be decoupled by using stars with different softness, namely, with a different number and length of arms, therefore deswelling degrees, as discussed later in the text.

In soft colloids or mixtures, a hybrid polymeric and colloidal rheological response can be typically detected, as in the case of previous star–linear polymer mixtures,^5^ grafted nanoparticle melts,^36^ or diblock copolymer micelles.^59^ The polymeric and the colloidal responses are characterized by two different stress relaxation mechanisms that occur on different time scales and proceed hierarchically. At high frequencies, the response is dominated by the polymer matrix, and the plateau modulus reflects the polymeric network response, and is estimated by taking into account only the free volume accessible to the chains.^40^ Once the linear chains relax, the dynamics are controlled by the colloidal star polymers, whose fingerprint is usually evident in the low-frequency region (colloidal response), typically <0.1 rad/s. This dual polymeric and colloidal response is also evident in the present investigated systems, see for example the mixture at *C*_L_ = 5 wt % in Figure 2A. However, with increasing linear polymer concentration, for example at *C*_L_ = 30 wt % (Figure 2A), the rheological response is dominated by the linear polymers, and a crossover between the moduli emerges at low frequencies. The colloidal response is here masked by the polymer dynamics and barely detectable in the probed frequency range. The mixtures exhibit the features of an ergodic viscoelastic liquid. The separation of the polymeric and colloidal responses is evident when the high-frequency (*G*_N_^0^) and the low-frequency (colloidal) (*G*_p_) plateau moduli, estimated from the relative minima in the loss factor (*G*″/*G*′), are plotted against the linear polymer volume fraction (Figure 2C). The polymeric (high-frequency) plateau modulus follows the expected trend for linear chains in a good solvent, *G*_N_^0^(ϕ) ∼ ϕ_L_^2.3^,^40^ whereas on the contrary, the colloidal (low-frequency) plateau clearly diverges from the linear polymer scaling.

To study this double transition in more detail and confirm the presence of this new re-entrance in the state diagram of equal-size star–linear polymer mixtures, we investigated a slightly lower star-polymer concentration (ϕ_s_ = 0.83). We also performed complementary creep experiments (see also Figure S2 in the Supporting Information) to extend the probed frequency window. A remarkable and even richer behavior of the mixtures is observed upon addition of linear chains at this ϕ_s_ (Figure 2B). Starting from a viscoelastic liquid in the absence of the linear polymer, the system evolves toward a critical gel (percolation threshold)^60^ upon increasing *C*_L_ (see data at *C*_L_ = 4.3 wt % in Figure 2B). Its dynamic moduli *G*′ and *G*″ overlap and follow a power-law dependence on frequency over a wide frequency range. Following ref (60), we can express the equivalent stress relaxation modulus at *C*_L_ = 4.3 wt % as *G*(*t*) = *S*_g_*t*^–*n*^, where *S*_g_ is the strength of the gel and *n* is the relaxation exponent (Figure 2D). When *n* assumes a value of 0 or 1, *S*_g_ represents the plateau modulus or the viscosity, respectively. In the present case, *n* was equal to 0.42, and the strength of the gel was 67 Pa s^0.42^. Although there are no universal values for *n* and *S*_g_, due to the nature of the gelling system investigated, our values are comparable to those reported in the literature for critical colloidal gels (0.4 < *n* < 1).^60−62^

The emergence of such criticality is universal when gelation is induced in an initially well-dispersed colloidal liquid, and it represents very important evidence for the progressive buildup of the gel structure for increasing *C*_L_. In contrast, this is the first time to our knowledge that such critical behavior is observed at such a high colloidal volume fraction (ϕ_s_ = 0.83): colloidal softness hampers gel formation in the range of volume fractions (ϕ_gel_) where gels of hard particles typically form, from a few percent up to the vitrification threshold ϕ_gel_ ≅ 0.58–0.60.^63^

A further increase in *C*_L_ drives the system to solid-like behavior at low frequencies, with the emergence of a low-frequency colloidal plateau modulus (see data at *C*_L_ = 6 wt % in Figure 2B). The latter assumes a value of about 40 Pa, yielding an apparent colloidal correlation length ξ_c_ =  = 48 nm, slightly larger than the colloidal radius (*R*_H_ = 39 nm). This suggests a non-interpenetrating condition for the stars. It is worth noting that for star-polymer glasses (in the absence of linear chains), where colloids are arranged in a cage-like fashion, ξ_c_ is typically only a fraction of the star radius and has been interpreted as the extent of the overlapping region between two neighboring stars.^64^ It is important to stress that the quantity ξ_c_ should not be confused with the mesh size of the polymer network ξ.

To further exclude the presence of caging we can exploit an alternative, yet equivalent, interpretation of the colloidal correlation length based on the analysis of glassy microgel suspensions by Cloitre et al.,^65^ where the plateau modulus is linked to the maximum displacement of a generic particle with radius *R*_H_ inside its topological cage. When a soft particle with radius *R*_H_ moves over a distance δ, it deforms elastically its neighbors, which in turn push it back inside the cage. The restoring energy that drives the particle back can be written as *G*_N_^0^δ^2^*R*_H_. When the latter equals the thermal energy, a maximum displacement δ is reached. This simple model was applied to the present star–linear polymer mixtures to estimate the maximum displacement of the constrained stars in the polymer matrix, yielding δ = 50 nm, that is, larger than the star hydrodynamic radius. By combining the two findings, ξ_c_ = 48 nm and δ = 50 nm, it is possible to assert that stars are not truly in a glassy state, but rather in a percolated network with reduced possibility to diffuse, due to the presence of the polymeric network and depletion attractions. We recall that in repulsive glasses, according to the Lindemann criterion,^64,66^ the maximum displacement is typically at most one tenth of the particle radius (about 4 nm here). We further recall that the pure star-polymer solution at this concentration (ϕ_s_ = 0.83) exhibits liquid-like behavior (Figure 2B). We conclude that caging must be excluded.

As already witnessed for the star–linear polymer mixtures at ϕ_s_ = 0.9, the progressive addition of linear chains to mixtures with ϕ_s_ = 0.83 gives rise to a re-entrant liquid (see data at *C*_L_ = 7.5 wt % in Figure 2B). However, the viscoelastic character of the mixtures and the role of the stars as outlined above are also evident here since the terminal flow regime is not attained over the examined frequency range. Indeed, the power-law behavior of the moduli (*G*′ ∼ ω^2^, *G*″ ∼ ω^1^) typical for a fully relaxed liquid is not observed even at the highest concentration investigated here (*C*_L_ = 30 wt %). Overall, whereas for mixtures with *C*_L_ below 7.5 wt % the rheological response is significantly affected by the presence of stars, at higher values of *C*_L_ the linear polymer chains dominate the linear viscoelastic spectra, however without fully suppressing the colloidal response.

We also remark that, unlike the case described in Figure 2A (ϕ_s_ = 0.9), where a dynamically arrested state is clearly attained over several decades in frequency upon addition of linear chains, for a slightly lower value of ϕ_s_ = 0.83, the concentration of star polymers is not high enough to promote a long-lasting arrested state. In fact, investigations at even lower star-polymer volume fractions (ϕ_s_ = 0.5, and ϕ_s_ = 0.7) displayed no dynamic arrest at any *C*_L_ (see Figure S4 in the Supporting Information). Nevertheless, the colloidal mode at low frequencies was still observable.

To further support the depletion-gel picture, exclude the possibility of chain-mediated bridging of stars, explore the influence of star-polymer softness, and decouple the dual deswelling-mesh size reduction effect, we also investigated nearly symmetric, in hydrodynamic size (see Table 1), S1114/L243 star–linear polymer mixtures. Here, the soft star was replaced with a HS-like star (S1114).

**Figure 3:**
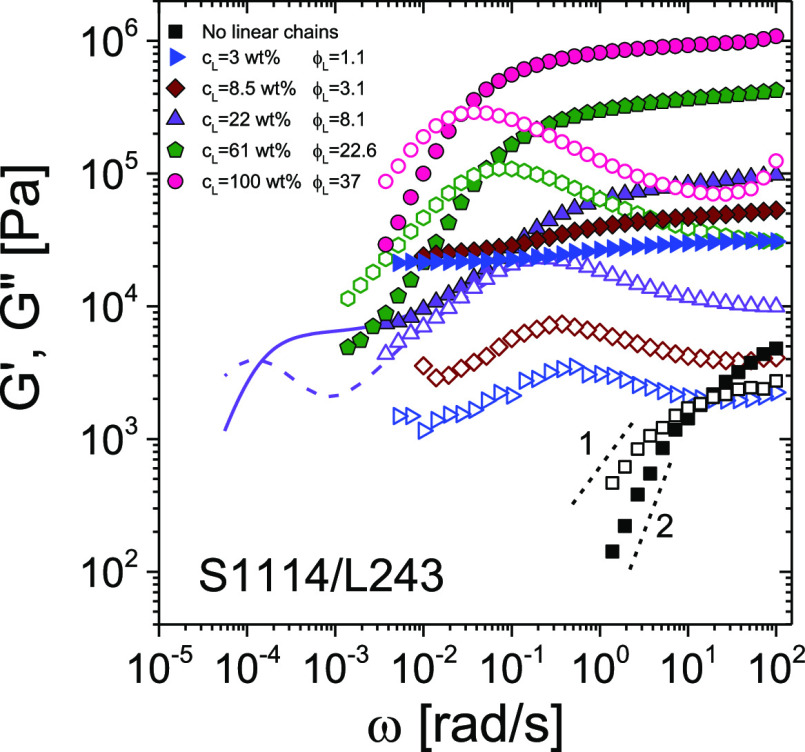
Linear viscoelastic spectra in terms of *G*′ (solid symbols) and *G*″ (open symbols) as a function of the oscillation frequency ω for the S1114–L243 mixtures. The squares represent the HS-like star-polymer (S1114) suspension at ϕ_HS_ = 0.8, below the glass transition (Table 1). The right-pointing triangles, diamonds, up-pointing triangles, pentagons, and circles correspond to mixtures at *C*_L_ = 3.0, 8.5, 22, 61, and 100 wt %, respectively. The circles correspond to pure linear chains. The lines are from creep data converted to dynamic moduli. The short-dashed lines highlight the terminal slope of fully relaxed viscoelastic fluids. All solutions were measured after steady-state conditions were reached (aging between 10^3^ and 10^5^ s).

The frequency-dependent *G*′ and *G*″ of S1114/L243 mixtures with a fixed volume fraction ϕ_HS_ (below ϕ_g_) of S1114 and increasing fractions of L243 are depicted in Figure 3. The dynamic frequency spectrum of the L243 melt is also included in the figure for comparison. The viscoelastic liquid S1114 star at ϕ_HS_ = 0.8 undergoes gelation upon addition of 3 wt % of L243. Note that at 3 wt %, the L243 chains are entangled (see the Materials section). This suggests again that the pivotal length scales for depletion are the size of the stars and the average correlation length (mesh size) of the polymer network, as also observed by Verma et al.^57^

As the concentration of linear chains is increased, the S1114 particles, which still conserve the deformability of soft colloids,^7^ deswell due to the osmotic pressure exerted by the linear chains. This is reflected in the significant reduction of the low-frequency moduli at *C*_L_ = 22 wt % of L243, and the remarkable separation between the polymeric and colloidal responses in the investigated frequency range.^5,36^ However, a further increase of *C*_L_ to 61 wt % does not lead to a fully relaxed viscoelastic liquid, as instead attained at *C*_L_ = 100 wt %. The HS-like stars still manifest their presence with the onset of a slow mode, which becomes progressively weaker in favor of terminal relaxation dynamics dominated by the linear chains.^7^

Therefore, the phenomenology encountered for the HS-like star–linear mixtures (S1114–L243) displays the same general features as the ones characterizing the softer star suspensions (S362–L1000), with a solid (gel) pocket of states separating the two seas of liquid states. Since the overall scenario remains unchanged, we speculate that an important role is played by the loss of efficiency of the depletion mechanism at very high *C*_L_ (due to the decrease in mesh size ξ) as this would intervene even in model HS systems.

**Figure 4:**
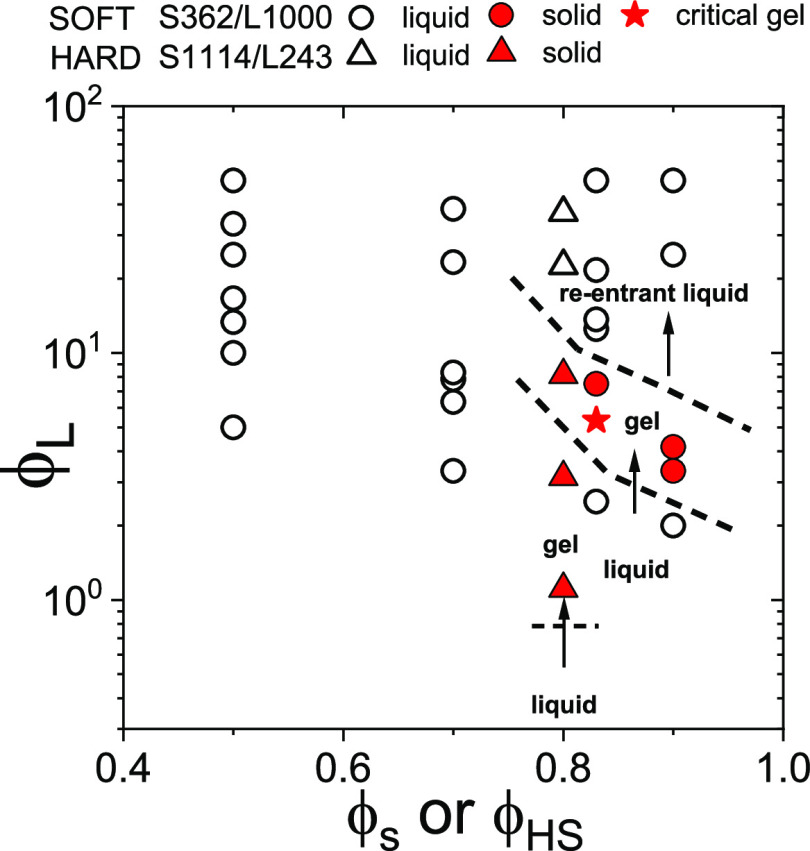
State diagram for equal-size star–linear polymer mixtures: S362–L1000 (circles and stars) and S1114–L243 (triangles) mixtures in the ϕ_L_–ϕ_s_ plane. The open symbols, filled stars, and filled circles indicate the liquid, critical gel, and solid states, respectively. The arrows and the dashed lines indicate the approximate location of the boundaries of the liquid-to-solid and the solid-to-re-entrant liquid transitions. It is worth recalling that ϕ_g_ is in the range 0.8–0.92 for S1114 and 1.5–2 for S362 (see Table 1).

To highlight more clearly the role of softness, we can proceed one step further and directly compare our data to determine whether the different star functionalities quantitatively tune such a re-entrant behavior. To this end, we compiled the rheological results discussed above in the form of a state diagram, which is depicted in Figure 4 in terms of linear chain volume fraction (ϕ_L_) and volume fraction of the stars (ϕ_s_). Rheological results for the mixtures at ϕ_s_ = 0.5 and ϕ_s_ = 0.7 are reported in the Supporting Information (Figure S4). The assignment of solid-like behavior is based on the linear viscoelastic spectra. In the diagram, we consider as viscoelastic solids those suspensions showing a storage modulus exceeding the loss modulus, at least down to 0.01 rad/s, meaning a structural relaxation time exceeding 100 s.^6,20,31,37,43^ An exception is represented by the mixture at ϕ_s_ = 0.83 and *C*_L_ = 4.3 wt %, that is, when critical gel behavior was observed.

As can be seen in Figure 4, the two sets of mixtures (S362–L1000 and S1114–L243) share the same qualitative behavior, but the extent of the solid-like pocket can apparently be tuned by the colloidal softness: the gel pocket is wider in the case of S1114–L243 mixtures, even though in this case the star number density is slightly lower than that characterizing the softer S362–L1000 systems, for which re-entrance is observed. We speculate that enhanced osmotic deswelling in the S362–L1000 mixtures reduces the extent of the solid-like behavior (see Figure 4), favoring the emergence of the re-entrant liquid at lower ϕ_L_. Conversely, depletion forces appear to be more effective in hard star suspensions^1^ that undergo gelation at much lower ϕ_L_, compared to the soft stars.

Hence, our rheological data shed light on a new aspect of the state diagram of soft colloids in the presence of depleting agents forming networks: the osmotic shrinkage of colloids due to the presence of linear chains and the loss of the depletion efficacy together give rise to a re-entrant liquid. We have shown that the addition of polymeric depletants has a dual effect on a liquid soft colloidal system with a fixed particle number density. Depletion attractions and osmotic deswelling impact the structural relaxation of the suspensions: the first contribution induces gel formation, while the second one leads to its melting.

**Figure 5:**
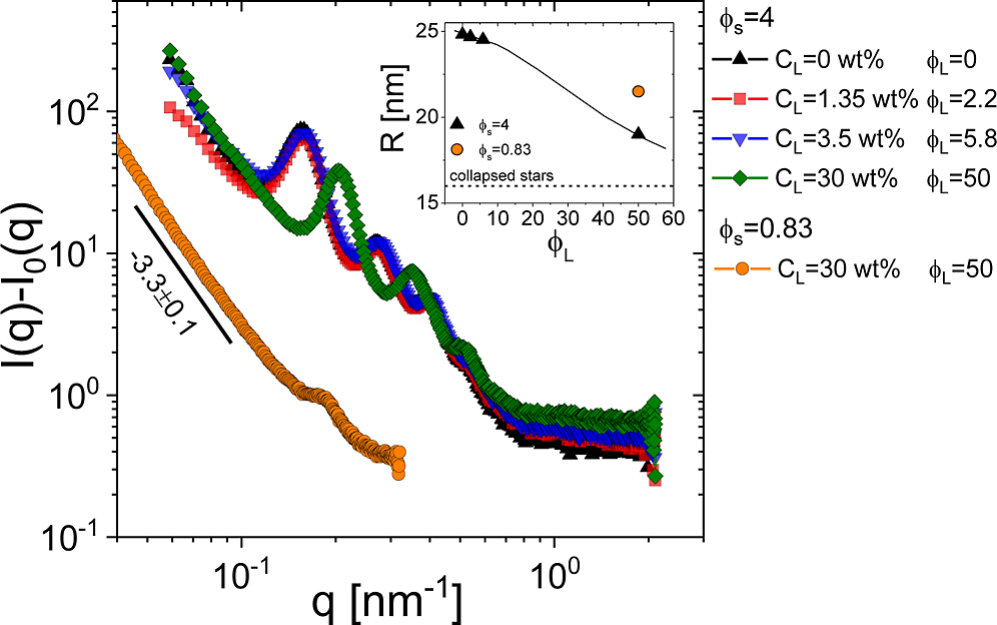
SAXS intensity [*I*(*q*) – *I*_0_(*q*)] for the S362–L1000 mixtures in squalene at fixed ϕ_s_ = 0.83 and ϕ_s_ = 4 and various linear polymer concentrations *C*_L_ (see the legend) as a function of the scattering wavevector *q*. The line through the ϕ_s_ = 0.83 data represents the best fit obtained with a power-law having an exponent of −3.3. This gives a surface fractal dimension equal to *d*_s_ = 2.7 ± 0.1 (see the main text). Inset: star radius (*R*) at ϕ_s_ = 0.83 and ϕ_s_ = 4 as a function of linear polymer volume fraction (ϕ_L_). *R* was estimated as π*a*/*q*_max_ where *q*_max_ is the scattering wavevector at the low-*q* intensity peak, and the coefficient *a* = 1.23 reflects a structure controlled only by two-body correlation.^52^ The horizontal dashed line represents the calculated radius of the totally collapsed S362 star,^31^ whereas the continuous line is a guide for the eye.

At the same time, star-polymer deswelling causes an increase in free volume, which facilitates the relaxation of linear chains. The SAXS data confirm this scenario and provide evidence for the central role of star deswelling, as demonstrated in Figure 5, which depicts the SAXS intensity *I*(*q*) as a function of the scattering wavevector for two star–linear polymer mixtures at various linear chain concentrations. The volume fractions of the pure star-polymer solutions are ϕ_s_ = 0.83 and ϕ_s_ = 4. The limited data set obtained at low star-polymer volume fractions reflects the lack of material available and a weak scattering signal. Nevertheless, two important messages can be drawn from this figure: (i) for the mixture at ϕ_s_ = 4, the structural peaks shift toward larger scattering wavevector as the concentration of linear chains is increased. This horizontal shift is remarkable at *C*_L_ = 30 wt % (see lozenges in Figure 5) and implies that the distance between scatterers (mainly the silicon-rich star cores, whose number density is fixed) decreases as *C*_L_ is increased. The low-*q* intensity peak gives access to the average distance between star cores, and if we consider that the stars are in contact due to depletion forces and that interpenetration is scarce (as expected for high-*f* stars in a good solvent^7^), a smaller effective radius can be computed for the (deswollen) colloidal stars as *R* = π*a*/*q*_max_. This is shown in the inset of Figure 5. Here, we may note that at *C*_L_ = 30 wt % and ϕ_s_ = 4, the size of the stars (18 nm) is not far from that corresponding to their calculated collapsed state (16 nm)^7,31,38^ and, most importantly, it is in excellent agreement with the star size computed from free volume considerations, as reported in ref (31) and the osmotic theory (see the Supporting Information). (ii) Shrinkage of the stars is also evident at lower number densities, as seen in Figure 5 for ϕ_s_ = 0.83 and *C*_L_ = 30 wt %. The effective star radius obtained from the observed correlation peak is about 21 nm, and by considering *R*_H_ = 39 nm (Table 1), it is possible to estimate a star shrinkage of nearly 50%. We recall that the rheology of the mixture at ϕ_s_ = 0.83 and *C*_L_ = 30 wt % (see Figure 2) clearly displayed viscoelastic liquid behavior with a barely detectable colloidal mode. Interestingly, the net attraction between stars arising from unbalanced osmotic forces is confirmed by the low-q power-law upturn *I*(*q*) ∼ *q*^–3.3±0.1^ in the SAXS spectrum for the ϕ_s_ = 0.83 and *C*_L_ = 30 wt %. We probed the surface fractal region^67,68^ in Fourier space, where *I*(*q*) ∼ *q*^–(6–*d*_s_)^, from which we obtained a surface fractal dimension *d*_s_ = 2.7 ± 0.1. In gels, *d*_s_ is a measure of the roughness between the colloid-rich and the colloid-poor regions, ranging between 2 (smooth surfaces) and 3 (infinitely rough surfaces). The obtained value therefore suggests the presence of very rough interfaces and the formation of highly porous structures. Consequently, our results indicate that these depletion gels of star polymers do not form bicontinuous networks with sharp interfaces between colloid-rich and colloid-poor regions. This is in sharp contrast with the gel structure of HS–polymer mixtures, in which the Porod scaling *I*(*q*) ∼ *q*^–4^, corresponding to *d*_s_ = 2, has been observed most commonly.^69^

Last but not least, we compared our shrinkage results from SAXS with the theoretical predictions based on Flory-type arguments,^5,26,30,53^ accounting for the osmotic, elastic, and interaction free energy (see Discussion and Figure S5 in the Supporting Information), obtaining very good agreement (within 10%).

This finding supports the evaluation of the star size via the analysis of the SAXS data and corroborates the existence of star polymers in close contact being at the origin of the gel-like response probed by linear rheology. A short note on the reduction of the effective volume fraction of the stars follows. Based on the values of the radii reported in the inset of Figure 5, the effective volume fraction decreases from ϕ_s_ = 4 (at ϕ_L_ = 0) to ϕ_s_ = 0.46 (at ϕ_L_ = 50). For the mixtures undergoing multiple transitions (Figure 2), the radius of the stars can be inferred from the viscoelastic spectra following the “chemical approach” (see the Supporting Information) described in ref (20). We obtain volume fractions ranging, respectively, from ϕ_s_ = 0.83 and ϕ_s_ = 0.9 at ϕ_L_ = 0 to ϕ_s_ = 0.15 and ϕ_s_ = 0.165 at ϕ_L_ = 50. Hence, it is not surprising that the rheology of the mixtures is highly affected by a drastic reduction of the volume effectively occupied by the colloidal component of the liquid (the stars), causing a drop of the stress response originating from the star-polymer network.

In closing, we emphasize again that the above analysis does not exclude the possible parallel action of the mechanism of suppression of depletion attraction, due to a smaller polymer mesh size for increasing concentrations, as already discussed.

## 4 Conclusions

In this work, we have shown that star-polymer suspensions (used as paradigms for soft colloids), below their overlap concentration, can undergo a liquid-to-gel transition upon addition of entangled linear homopolymer chains having nearly the same hydrodynamic size. The origin of gelation is attributed, as in the case of hard sphere–linear polymer mixtures, to depletion forces arising from the asymmetry between the size of the star polymer and the correlation length of the linear chain matrix. Remarkably, a further increase in the concentration of linear chains gives rise to an unprecedented re-entrant liquid. This phenomenon is promoted by the gain in free volume due to the deswelling of the stars (osmotic shrinkage) for increasing linear chain concentrations (acting as osmotic compressors). The shrinkage effect is also supported by structural results obtained from SAXS experiments. Indeed, a clear shift observed in the main peaks of the structure factor of star-polymer suspensions, in the presence of linear additives, reflects star deswelling. A parallel effect contributing to the observed re-entrance is the fact that the reduced mesh size at higher polymer concentrations leads to a smaller range of depletion attractions, hence making them less probable.

As the concentration of star polymers is reduced to ϕ_s_ = 0.83, further departing from nominal overlap, the sensitivity of the rheological response to the addition of linear polymer increases. A critical-gel condition precedes a solid-like response at low frequencies (solid-like pocket), and a correlated viscoelastic liquid with colloidal response is detected. In such mixtures, the concentration of soft colloids is not sufficiently large to promote an arrested state extending over several decades of frequency, contrary to what was observed at ϕ_s_ = 0.9. The rheological data were used to construct a dynamic state diagram of polymer versus star concentration for equal-size star–linear polymer mixtures, which significantly differs from those of respective mixtures with large size asymmetry,^5^ or star–star,^8,9^ star–hard sphere,^1,7^ and HS–hard sphere^11^ mixtures investigated so far. None of these mixtures, all with large size asymmetries, displayed re-entrant melting of a dynamically arrested state. The unique phenomenology that we encountered in the present work largely depends on the depletant-to-colloid size ratio, dynamic properties of the depletants, colloidal softness, and solvency conditions. Contrarily to what we have reported here, largely asymmetric star–linear polymer mixtures can experience a completely different gel-to-liquid-to-gel multiple transition that has been previously discussed.^20^ Our volume fraction estimation for the stars further suggests that the reduction in size of soft colloids due to osmotic compression is very relevant for the rheology of mixtures, and it emphasizes once again the importance of determining the volume fraction of soft colloids especially in the presence of osmotic compressors.

We conjecture that moving from good to poor solvent condition would cause the linear chains to adsorb on the stars, suppressing depletion and possibly driving a gelation mediated by linear chains, the latter causing energetic bridging between the colloids (the stars here). Both species (linear and stars) would experience solvophobic attractions when in close contact with another suspended component of the mixtures: star–star, linear–linear, and star–linear interactions would include an attractive term that could bring to microphase separation and progressively stabilize the gel phase. Hence, we would expect a totally different outcome that could be corroborated in future studies.

Blending colloidal systems with different softness levels and molecular structures revealed a simple, yet elegant way to control phase transitions, as well as the flow properties of suspensions.

## Supporting Information Available

DLS analysis results for sample L243; creep compliance and conversion into dynamic moduli; shear rheology of star (S362)–linear (L1000) mixtures at ϕ_s_ = 0.5 and ϕ_s_ = 0.7 at various linear chain concentrations; and Flory’s prediction on the shrinkage of a star polymer in a solution of linear homopolymer chains (PDF)
