# Supplementary material for: Gelation and Re-entrance in Mixtures of Soft Colloids and Linear Polymers of Equal Size
Source: Macromolecules. 2023 Feb 22;56(5):1818–27. doi: 10.1021/acs.macromol.2c02491 (PMC10019458; doi:10.1021/acs.macromol.2c02491)
Supplement: Supplementary file 1 — ma2c02491_si_001.pdf [file ma2c02491_si_001.pdf]

Supplementary Material

Gelation and Re-entrance in Mixtures of Soft Colloids and Linear Polymers of Equal Size

Daniele Parisi,[1,2,3*] Domenico Truzzolillo,[4*] Ali H. Slim,[5] Philippe Dieudonné-George,[4] Suresh Narayanan,[6] Jacinta C. Conrad,[5] Vishnu D. Deepak,[7] Mario Gauthier,[7] and Dimitris Vlassopoulos[1,2]

[1]FORTH, Institute of Electronic Structure and Laser, 70013 Heraklion, Crete, Greece

[2]Department of Materials Science and Technology, University of Crete, 70013 Heraklion, Crete, Greece

[3]Department of Chemical Engineering, Product Technology, University of Groningen, Nijenborgh 4, 9747 AG Groningen, The Netherlands

[4]Laboratoire Charles Coulomb (L2C), UMR 5221 CNRS Université de Montpellier, Montpellier, France.

[5]Department of Chemical and Biomolecular Engineering, University of Houston, Houston, Texas 77204-4004, United States

[6]Advanced Photon Source, Argonne National Laboratory, Argonne, IL 60439, United States

[7]Department of Chemistry, University of Waterloo, Waterloo, Ontario N2L 3G1, Canada

*corresponding authors: domenico.truzzolillo@umontpellier.fr
d.parisi@rug.nl

Table of Contents

Dynamic light scattering (DLS) measurements of L243 linear polymer in squalene *2*

Linear viscoelasticity: Plateau modulus, creep conversion, and frequency response of the ϕ_S = 0.5 and ϕ_S = 0.7 mixtures *3*

On osmotic shrinkage: Flory-type approach *7*

References *10*

Dynamic light scattering (DLS) measurements of L243 linear polymer in squalene

DLS measurements were performed using the photon correlation spectroscopy technique[1]. The intensity autocorrelation function $G(q,t) = \langle I(q,t)I(q)\rangle/|I(q)|^2$ is obtained over a broad range of lag times ($10^{-7} - 10^3$ s) at different scattering wavevectors q, with an ALV-5000 goniometer/correlator setup (ALV, Germany) equipped with a Nd:YAG laser at wavelength λ = 532 nm (Oxxius, France). The scattering wavevector is computed as $q = (4\pi n/\lambda)\sin(\theta/2)$ where n and θ are the refractive index of the solvent and the scattering angle, respectively. Measurements were performed in the co-polarized (VV) geometry under homodyne beating conditions. The intermediate scattering function C(q,t) probing concentration fluctuations was extracted from the experimental intensity autocorrelation function acquired with the photomultiplier tube, $G(q,t):C(q,t)=\left[\frac{G(q,t)-1}{A}\right]^{1/2}$, where A ≤ 1 is an instrumental coherence factor, equal to 0.6 for the aforementioned setup.

The experiments were performed under dilute conditions for characterization purposes, and a single exponential relaxation process was observed. By applying the Stokes-Einstein-Sutherland relation $D=\frac{k_B T}{6\pi R_H \eta_{squalene}}$, which relates the diffusion coefficient with the hydrodynamic size of the particles, the thermal energy $k_B T$, and the viscosity of the suspending medium $\eta_{squalene}$, the hydrodynamic radii were extracted. All the measurements were performed at 20 ℃. Figure S1 below shows the DLS results for sample L243 in squalene.

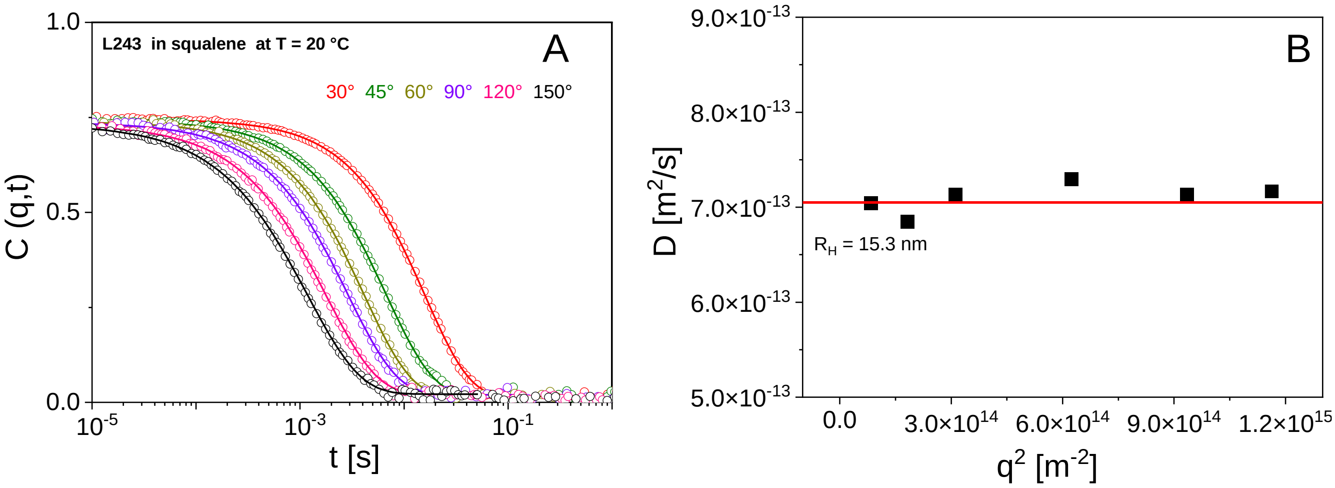

Figure S1. (A) Normalized intermediate scattering function C(q,t) for sample L243 in squalene at 3×10^{-4} g/ml. Each color corresponds to a different scattering angle (red: 30°, green: 45°, dark yellow: 60°, purple: 90°, pink: 120°, black: 150°). The solid lines represent fits to single exponential functions. (B) Diffusion coefficient as a function of q^2 for the L243 sample. From this plot, the hydrodynamic radius (R_H) was extracted using the extrapolated value of D at $q^2 = 0$, and the Stokes-Einstein- Sutherland equation for the diffusion of spherical particles in a medium.

Linear viscoelasticity: creep conversion, and frequency response of the $\phi_s = 0.5$ and $\phi_s = 0.7$ mixtures

Creep compliance measurements for a representative S362–L1000 mixture at $\phi_s = 0.83$ and $C_L = 7.5$ wt% are provided in Fig. S2A. The creep compliance was always measured at least at two different shear stresses (see Figure S2) to ensure that the measurements were in the linear viscoleastic response region. Deviations within 10% between the data sets were considered acceptable, given the difficulty of such experiments with aging systems. Indeed, the structure of colloid–polymer mixtures may change over time[2], making the creep experiments more difficult to reproduce. Note that the first two time decades, where the known "ringing" phenomenon occurs[3], are usually not considered for creep data conversion. Nevertheless, the conversion of this region is still shown for completeness. Figure S2B shows conversion of the creep compliance data into dynamic moduli as a function of the oscillation frequency. Dynamic moduli obtained from small amplitude oscillatory shear measurements are also shown in the same Figure as symbols, to validate the creep data and their conversion.

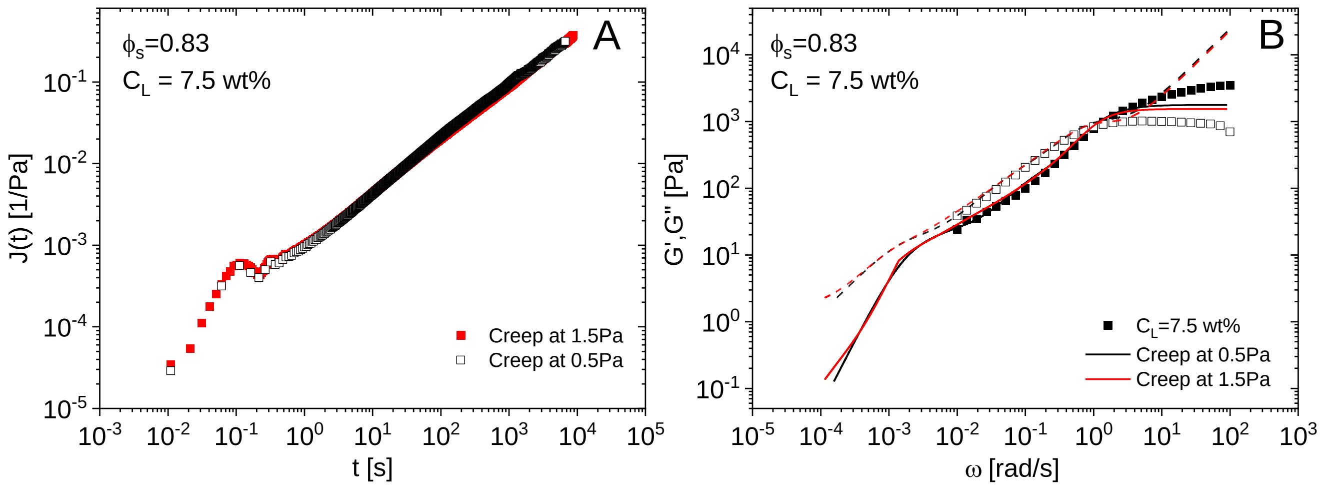

Figure S2. (A) Creep compliance as a function of time for a S362–L1000 mixture at $\phi_s = 0.83$ and $C_L = 7.5$ wt%, measured at two different shear stresses: 1 Pa (blue symbols) and 1.5 Pa (black symbols). (B) Storage G' (solid symbols) and loss G" (open symbols) moduli as a function of the oscillatory frequency ω for a S362–L1000 mixture at $\phi_s = 0.83$ and $C_L = 7.5$ wt%. The data obtained from small amplitude oscillatory shear are reported as symbols, whereas the lines represent the creep conversion into storage modulus (solid line) and loss modulus (dashed line) at 1 Pa (black lines) and 1.5 Pa (red lines). Experiments performed at 20 °C.

A short description of the creep conversion follows in order. The shear creep compliance J(t) should be first converted into the continuous spectrum of retardation times, L(τ). J.D. Ferry proposed the exact interrelation between the J(t) and L(τ) functions[4]:

$$J(t) = J_g + \int_{-\infty}^{\infty} L\left(1 - e^{-\frac{t}{\tau}}\right) d\ln(\tau) + t/\eta_0 \qquad \text{(S1)}$$

with J_g being an instantaneous compliance added to allow for the possibility of a discrete contribution with t = 0. η_0 is the zero-shear viscosity. The retardation spectrum enables the determination of the dynamic compliance moduli:

$$J'(\omega) = J_g + \int_{-\infty}^{\infty} \left[\frac{L}{(1+\omega^2\tau^2)}\right] d\ln(\tau) \qquad \text{(S2)}$$

$$J''(\omega) = \int_{-\infty}^{\infty} \left[\frac{L\omega\tau}{(1+\omega^2\tau^2)}\right] \mathrm{dln}(\tau) + 1/(\omega\eta_0) \qquad \text{(S3)}$$

The latter can be directly converted into dynamic moduli by means of:

$$J'(\omega) = \frac{G'(\omega)}{G'^2(\omega) + G''^2(\omega)} \tag{S4}$$

$$J''(\omega) = \frac{G''(\omega)}{G'^2(\omega) + G''^2(\omega)} \tag{S5}$$

The challenge is then the conversion between the shear creep compliance and the retardation spectrum, and this into dynamic compliance. Determining L(τ) from J(t) represents a mathematically ill-posed problem, as various L(τ) functions can lead to the same J(t). The most renowned mathematical method is the regularization method of Tikhonov[5]. In the present case, the relation between the physically interesting function and the experimental data is given by nonlinear integral equations, further complicating the math. Weese proposed the so-called nonlinear regularization method[6]; a mathematical treatment that allows for the determination of a physically interesting function, described by a nonlinear integration function. Weese implemented his model in a software named NLREG. In our case, we implemented the mathematical treatment in Mathematica®. This method is quite accurate, and the only significant source of error is the experimental error in measuring J(t). Figure S3 below shows the retardation spectrum at the two different stresses shown in Figure S2A, including error bars (within the symbol size).

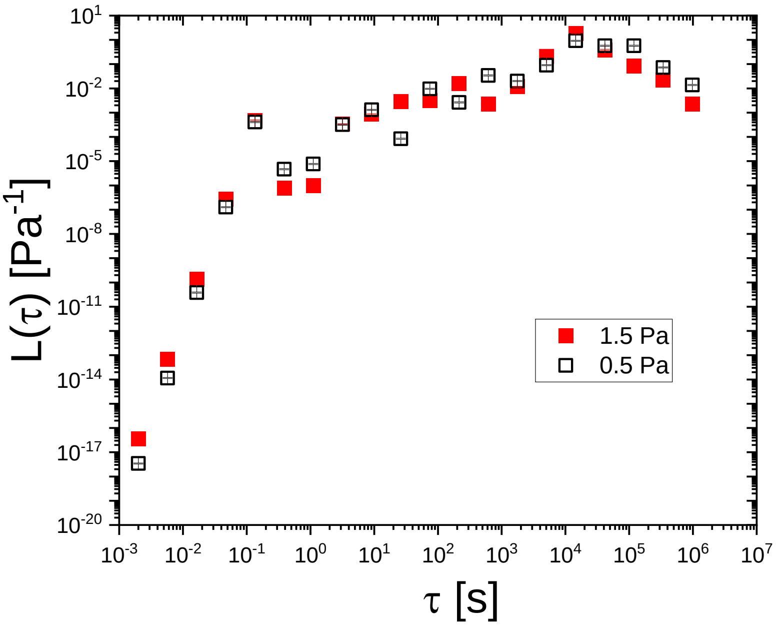

Figure S3. Retardation spectrum of the data shown in Figure S2A, obtained from the method proposed by Weese[6].

Fig. S4 depicts the linear viscoelastic spectra in terms of storage (G') and loss (G") moduli as a function of the oscillatory frequency for the S362–L1000 mixture at fixed $\phi_s = 0.5$ (left panel) and $\phi_s = 0.7$ (right panel) for increasing C_L. Note that, in contrast to the mixtures reported in the main text, no gelation was observed at any C_L value, albeit the colloidal mode was still observable at low frequencies.

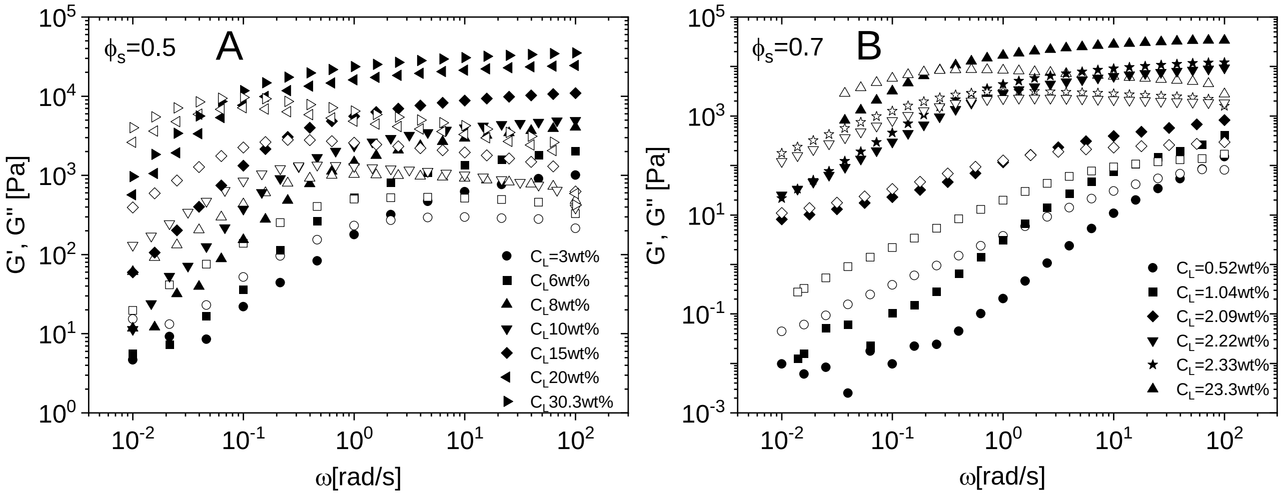

Figure S4. G' (solid symbols) and G" (open symbols) as a function of ω for the S362–L1000 mixture at fixed $\phi_s = 0.5$ (panel A) and $\phi_s = 0.7$ (panel B) for increasing C_L. Experiments performed at 20 °C.

On osmotic shrinkage: Flory-type approach

By using Flory-type arguments concerning the size of a star in a bath of linear polymer chains[7–10], it is possible to estimate the shrinkage of the star and the gain in free volume due to the osmotic pressure exerted by the linear chains onto the star. Firstly, the shrinkage of the star was calculated through the osmotic theory[7–10]. The free energy cost of inserting a star polymer with radius R in a solution of homopolymer linear chains corresponds to the mechanical work that needs to be done to create a spherical cavity within the solution:

$$\beta F_{os}(R) = \frac{4\pi}{3} R^3 a^3 \beta \Pi(\phi_L) \qquad \text{(S6)}$$

where $\beta = 1/k_BT$, *a* is the monomer radius (0.5 nm[11,12]), and $\Pi(\phi_L)$ the osmotic pressure exerted by the polymer matrix and is written as

$$\beta \Pi(\phi_L) = C_L[1 + P(\phi_L)] \qquad \text{(S7)}$$

with

$$P(x) = \frac{1}{2} x\, exp\left\{\frac{1}{4}\left[\frac{1}{x} + 1 - \frac{1}{x}\ln(x+1)\right]\right\} \qquad \text{(S8)}$$

and

$$x(\phi_L) = \frac{1}{2}\phi_L\, \pi^2 \left[1 - \frac{1}{4}\left(\ln 2 + \frac{1}{2}\right)\right] \qquad \text{(S9)}$$

where R represents the radius of the spherical cavity, which does not necessarily coincide with the size of the star R_s because of the penetrability of the stars. Camargo and Likos[13] have shown that a chain can penetrate a star up to a distance $\sigma = 4/3R_s = R$, which is the corona size of the star. It follows that $R = bR_s = 1.3R_s$. This numerical prefactor is essentially independent of the star/linear ratio, provided that it remains larger than 1. However, as in the present case, when the stars and linear chains are comparable in size, b must attain larger values because the chains would rather surround the stars than penetrate them. In the present work b = 1.7 was used, as suggested by Wilk *et al.*[8] when stars and linear chains have a comparable size.

The osmotic pressure is not the only contribution to the free energy. Associated with a star of radius R_s of functionality f and degree of polymerization N_s, the elastic and interaction free energies (excluded volume) are respectively given by

$$\beta F_{el}(R) = \frac{3}{2}\frac{afR_s^2}{N_s} \qquad \text{(S10)}$$

and

$$\beta F_{int}(R) = \frac{a^3\text{v}(fN_s)^2}{2R_s^3} \qquad \text{(S11)}$$

where v is the excluded volume parameter in reduced units, which has been given the value 1, corresponding to good solvency conditions[7,8]. The extent of shrinkage of the stars due to the presence of linear chains is determined as the value at which the overall free energy reaches a minimum. Thus, minimizing the free energy with respect to R_s the following result is obtained:

$$\frac{3afR_s}{N_s} + 4\pi b^3 R_s^2 a^3 C_L\left[1 + P\left(\phi_L\right)\right] - \frac{3a^3\text{v}(fN_s)^2}{2R_s^4} = 0 \qquad \text{(S12)}$$

By defining the overlap concentration as $C_L^* = a^{-3}N_L^{-3v}$[7,8] with v = 3/5 as the Flory exponent and N_L representing the degree of polymerization of the linear chains, and using the scaling relation for the size ratio between linear chains and stars (δ) as[10] $\delta = \left(\frac{1}{f^{1/5}}\right)\left(\frac{N_L}{Ns}\right)^v$, equation S12 becomes

$$\frac{3afR_s}{N_s} + 4\pi b^3 R_s^2 \delta^{-3} N_s^{-3v} f^{-3/5} \phi_L\left[1 + P\left(\phi_L\right)\right] - \frac{3a^3\text{v}(fN_s)^2}{2R_s^4} = 0 \qquad \text{(S13)}$$

The effective volume fraction of chains and the size of stars can be self-consistently evaluated as described in Ref. 7 for different star concentrations.

Results are reported as the shrinkage factor $g(\phi_L) = R_s(\phi_L)/R_{H,0}$ against ϕ_L with f = 362, N_s = 1240, N_L = 19600 and δ = 5 in Figure S5. $R_{H,0}$ is the hydrodynamic radius of the star in the absence of linear chains. Along with the theoretical predictions, are also reported the calculated shrinkage values (see Ref. 14) and the spacing of the scatterers determined from the SAXS measurements (see main text). Good agreement was observed between the calculated values, theoretical predictions and the SAXS data, further corroborating the osmotic shrinkage effect of the stars exerted by the star themselves and the linear polymer chains.

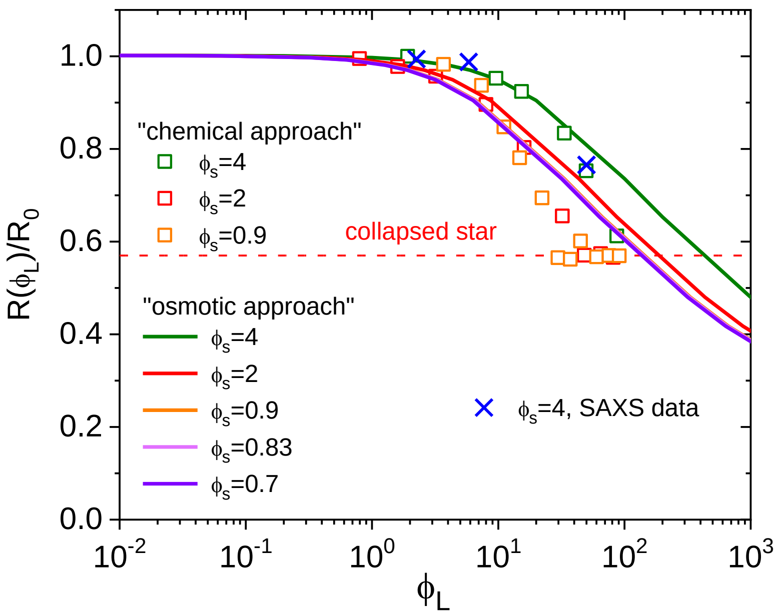

Figure S5. Osmotic shrinkage expressed as the ratio between the radius of the stars $R(\phi_L)$ at non-zero ϕ_L and its value R_0 in absence of chains as a function of the volume fraction of linear polymer chains ϕ_L. The lines represent the theoretical prediction, whereas the square symbols are the calculated values discussed in Ref. 14 (chemical approach). Predictions for ϕ_s=0.7, 0.83, 0.9 do not show any difference within the numerical precision of our code. The cross symbols were extracted from the SAXS data (see Figure 5 in the main text and relevant discussion). The dashed red line represents the calculated size of the fully shrunken star, which can be seen as the radius of a sphere containing fN_a close-packed monomers, where f is the star functionality and N_a the Kuhn degree of polymerization of one arm[14].

Table S1 below provides a comparison of the star radius for the S362 suspensions at various ϕ_s by two different approaches: 1) chemical approach[14], 2) and SAXS measurements.

Table S1. S362 estimated radius at various ϕ_s.

ϕ_s	chemical approach[14]	$d_{cc}/2 = R = \pi a/q_{max}$
0.9	38.3	
2	32.4	33.0
3		27.0
4	27.7	24.8
5		23.3

References

(1) Berne, B. J.; Pecora, R. *Dynamic Light Scattering: With Applications to Chemistry, Biology, and Physics*; Courier Corporation, **2000**.

(2) Truzzolillo, D.; Vlassopoulos, D.; Munam, A.; Gauthier, M. Depletion Gels from Dense Soft Colloids: Rheology and Thermoreversible Melting. *Journal of Rheology* **2014**, *58* (5), 1441–1462.

(3) Ewoldt, R. H.; McKinley, G. H. Creep Ringing in Rheometry or How to Deal with Oft-Discarded Data in Step Stress Tests! *Rheol. Bull* **2007**, *76* (4).

(4) Ferry, J. D. *Viscoelastic Properties of Polymers*; John Wiley & Sons, **1980**.

(5) Nashed, M. Z. The Theory of Tikhonov Regularization for Fredholm Equations of the First Kind (Cw Groetsch). *Siam Review* **1986**, *28* (1), 116–118.

(6) Weese, J. A Regularization Method for Nonlinear Ill-Posed Problems. *Computer Physics Communications* **1993**, *77* (3), 429–440.

(7) Truzzolillo, D.; Vlassopoulos, D.; Gauthier, M. Osmotic Interactions, Rheology, and Arrested Phase Separation of Star–Linear Polymer Mixtures. *Macromolecules* **2011**, *44* (12), 5043–5052.

(8) Wilk, A.; Huißmann, S.; Stiakakis, E.; Kohlbrecher, J.; Vlassopoulos, D.; Likos, C. N.; Meier, G.; Dhont, J. K. G.; Petekidis, G.; Vavrin, R. Osmotic Shrinkage in Star/Linear Polymer Mixtures. *The European Physical Journal E* **2010**, *32* (2), 127–134.

(9) Flory, P. J. The Configuration of Real Polymer Chains. *The Journal of Chemical Physics* **1949**, *17* (3), 303–310.

(10) Likos, C. N. Effective Interactions in Soft Condensed Matter Physics. *Physics Reports* **2001**, *348* (4–5), 267–439.

(11) Likos, C. N.; Löwen, H.; Poppe, A.; Willner, L.; Roovers, J.; Cubitt, B.; Richter, D. Ordering Phenomena of Star Polymer Solutions Approaching the Θ State. *Physical Review E* **1998**, *58* (5), 6299–6307.

(12) Li, X.; Ma, X.; Huang, L.; Liang, H. Developing Coarse-Grained Force Fields for Cis-Poly (1, 4-Butadiene) from the Atomistic Simulation. *Polymer* **2005**, *46* (17), 6507–6512.

(13) Camargo, M.; Likos, C. N. Unusual Features of Depletion Interactions in Soft Polymer-Based Colloids Mixed with Linear Homopolymers. *Physical review letters* **2010**, *104* (7), 078301.

(14) Parisi, D.; Truzzolillo, D.; Deepak, V. D.; Gauthier, M.; Vlassopoulos, D. Transition from Confined to Bulk Dynamics in Symmetric Star–Linear Polymer Mixtures. *Macromolecules* **2019**, *52* (15), 5872–5883.
